# Post hoc analysis of initial treatments and control status in the INITIAL study: an observational study of newly diagnosed patients with asthma

**DOI:** 10.1186/s12890-020-1069-2

**Published:** 2020-04-09

**Authors:** Jiangtao Lin, Xiuhua Fu, Ping Jiang, Weidong Song, Xiaoyun Hu, Zhijun Jie, Chuntao Liu, Zhengguang He, Xiangdong Zhou, Huaping Tang

**Affiliations:** 1 Department of Pulmonary and Critical Care Medicine, China-Japan Friendship Hospital, No.2 Yinghua East Street, Chaoyang District, Beijing, 100029 China; 2 Department of Pulmonary and Critical Care Medicine, The Affiliated Hospital of Inner Mongolia Medical University, Hohhot, 010050 China; 3 Department of Respiratory Diseases, Tianjin First Center Hospital, Tianjin, 300192 China; 4 Department of Respiratory Diseases, Peking University Shenzhen Hospital, Shenzhen, 518035 China; 5 Department of Respiratory Diseases, First Hospital of Shanxi Medical University, Taiyuan, 030001 China; 6 Department of Respiratory Diseases, The Fifth People’s Hospital of Shanghai, Fudan University, Shanghai, 200240 China; 7 Department of Respiratory Diseases, West China Hospital, Sichuan University, Chengdu, 610041 China; 8 Department of Respiratory Diseases, Suining Central Hospital, Suining, 629000 China; 9 Department of Respiratory Diseases, The First Hospital Affiliated to AMU (Southwest Hospital), Chongqing, 400030 China; 10 Department of Respiratory Diseases, Qingdao Municipal Hospital, Qingdao, 266000 China

**Keywords:** Global initiative for asthma, Inhaled corticosteroid, Leukotriene receptor antagonist, Long-acting β_2_ agonist, Symptom control

## Abstract

**Background:**

The 12-week, multicentre, observational INITIAL study (NCT02143739) assessed asthma severity in newly diagnosed Chinese patients.

**Methods:**

Post hoc analysis of medication combinations prescribed per routine clinical practice at baseline, and the impact on control levels evaluated using 2012 vs 2018 Global Initiative for Asthma (GINA) criteria.

**Results:**

In total, 4491 patients were included in the analysis. At baseline, intermittent, mild, moderate and severe asthma was reported in 3.9, 12.0, 22.6 and 61.6% of patients, respectively. Most patients (90.2%) were prescribed inhaled corticosteroid/long-acting β_2_ agonist (ICS/LABA). ICS/LABA plus ≥1 additional medication(s) was prescribed to 66.7% of patients, with leukotriene receptor antagonist (LTRA, 54.7%) being the most common additional medication. Distribution of ICS/LABA vs ICS/LABA+LTRA was comparable in patients with intermittent (3.2% vs 3.0%), mild (11.5% vs 9.7%), moderate (21.2% vs 19.9%) and severe asthma (64.1% vs 67.4%). Control levels among patients using ICS/LABA+LTRA vs ICS/LABA were comparable using GINA 2012 and lower using GINA 2018 criteria.

The proportion of patients using ICS/LABA+LTRA vs ICS/LABA with intermittent, mild, moderate and severe asthma controlled at Week 12 (using GINA 2012) were 78.1% vs 80.0, 86.5% vs 85.8, 78.5% vs 71.3, and 59.6% vs 61.8%, respectively. Using GINA 2018 criteria proportions were 86.8% vs 95.9, 86.1% vs 93.2, 82.1% vs 85.3, and 71.9% vs 77.6%, respectively.

**Conclusions:**

Asthma control was not improved by adding LTRA to ICS/LABA and may have been unnecessary for some newly diagnosed patients. These findings were irrespective of the GINA criteria (2012 vs 2018) used and baseline severity.

## Background

Two pivotal long-term goals of asthma management are symptom control and risk reduction [1]. Evaluation of symptom control constitutes the basis of treatment decisions in a continuous asthma management cycle composed of assessment, treatment adjustment, and response review recommended by the Global Initiative for Asthma (GINA) [1, 2]. Criteria used to assess current clinical control have evolved from a combination of symptoms and lung function (peak expiratory flow [PEF] or forced expiratory volume in 1 s [FEV_1_]) endorsed by GINA 2012 [1] to symptoms alone in GINA 2014 [3] and subsequent GINA updates [2]. The removal of lung function from assessment of asthma control is based on the rationale that although still important in predicting risk of exacerbations [4, 5], lung function testing results, i.e. spirometry, sometimes provide equivocal or even little utility for determining the level of clinical control [6, 7]. Valid tools that can be used to assess symptom control include simple screening tools (e.g. the 4-item questionnaires endorsed by GINA) [1], categorical symptom control tools (e.g. Royal College of Physicians Three Questions’ Tool) [8], and numerical tools (e.g. Asthma Control Questionnaire [ACQ]) [9].

Inhaled corticosteroids (ICS) with or without long-acting β_2_ agonist (LABA) has been recommended by GINA as the mainstay initial controller treatment, and leukotriene receptor antagonist (LTRA) may be considered as an alternative option or add-on medication at step 2 to 4 [2]. LTRA belongs to anti-leukotrienes and renders anti-inflammatory as well as bronchodilating effects via inhibiting leukotriene receptor or altering leukotriene production. The oral route of administration gives this class of medications an advantage, and they may bring particular benefits for patients with asthma and allergic rhinitis in terms of relieving symptoms and preventing exacerbations [10]. However, the efficacy of LTRA for asthma control remains controversial, with most evidence so far favouring ICS over LTRA as the preferred maintenance therapy for asthma [11]. In China, a survey involving two provinces showed that LTRA was frequently prescribed in combination with ICS/LABA for patients with severe asthma (87.5% [49/56]) [12].

The INITIAL study (NCT02143739) was a 12-week, multicentre, prospective, observational study of patients with asthma comprising 45 centres across Northern and Southern China [13]. The study aimed to assess asthma severity among newly diagnosed patients as well as their prescribed medications and response to treatment. In the primary analysis of INITIAL data, we observed a high rate of LTRA prescription at baseline (62.1% [2788/4491]), especially in patients with severe asthma (ICS/LABA plus LTRA, 67.4% [1654/2455]; LTRA without ICS/LABA, 35.5% [118/332]) [13]. The current analysis was conducted to further investigate the medications and medication combinations prescribed at baseline during the INITIAL study, with a focus on ICS/LABA and ICS/LABA plus LTRA, and to determine the impact of the change in asthma control criteria between the Global Initiative for Asthma (GINA) 2012 [1] and 2018 guidelines [2].

## Methods

This was a post hoc analysis of a 12-week, multicentre, prospective, observational study performed in patients with asthma from 45 centres across Northern and Southern China between June 2014 and September 2016 (INITIAL, NCT02143739). The protocol, full details of the study population and overall results have previously been reported [13]. Methods specific to this post hoc analysis are briefly described below.

### Patients

Eligible patients were aged ≥18 years, had newly diagnosed asthma without exacerbations within 2 weeks and had not used ICS in the 3 months prior to enrolment. Diagnosis of asthma was made based on lung function test (performed for all patients, airflow limitation, reversibility, and variability based on FEV1 or PEF help confirm the diagnosis), the gold standard for asthma diagnosis, and based on symptoms typical of asthma (i.e. recurrent breathlessness, wheezing, cough, and chest tightness, often triggered by allergens, cold, physical or chemical irritations, viral infection, or exercise; wheezing sound and prolonged respiratory phases during flare-ups; alleviation of symptoms spontaneously or upon treatment; exclusion of other possible diseases that have similar symptoms), in accord with criteria recommended by Chinese guidelines for the prevention and management of bronchial asthma (2008, [14]). Key exclusion criteria were being diagnosed with COPD and having asthma exacerbations within 2 weeks of study inclusion.

### Medications

Any medications were prescribed as per routine clinical practice at baseline, Week 4, and Week 8 (with no additional monitoring or diagnostic procedures); treatment decisions were not part of the INITIAL study.

### Asthma severity and control

At baseline, patients were screened, and GINA-defined asthma severity [15] and control were assessed [1, 2]. Patient-reported outcomes were assessed using the Asthma Control Questionnaire (5-item version; ACQ-5) [16]. GINA asthma control status and ACQ-5 were assessed at Weeks 4, 8 and 12.

### Objectives of this post hoc study

The objectives of the post hoc analysis were to reveal medications prescribed at baseline for asthma, to assess asthma control levels at Week 12 based on GINA 2012 vs GINA 2018 criteria, and to investigate the impact of medications on asthma control.

### Statistical analysis

Descriptive statistics were used in this post hoc analysis. Qualitative variables were described by absolute counts and percentages. No inferential statistics were used.

## Results

### Patients

This post hoc analysis included all 4492 patients in the full analysis set (FAS), and overall, 3587 (74.5%) completed the study (Table 1) [13].

**Table 1:** Patient characteristics

		*N* = 4492 n (%)
Age (years)^a^	< 30	850 (19.0)
	30–60	3077 (68.8)
	> 60	543 (12.2)
Sex^b^	Male	1819 (40.5)
	Female	2672 (59.5)
Asthma history^b^	Yes	293 (6.5)
	No	4168 (92.8)
	Unknown	30 (0.7)
Smoking status^b^	Never	3381 (75.3)
	Ever	635 (14.1)
	Current	475 (10.6)
Area of residence^b^	Urban	3208 (71.4)
	Rural	1283 (28.6)
Allergy history^b^	Yes	992 (22.1)
	No	2832 (63.1)
	Unknown	667 (14.9)
GINA 2006 severity^a^	Intermittent	173 (3.9)
	Mild	538 (12.0)
	Moderate	1013 (22.6)
	Severe	2767 (61.6)

*GINA* Global Initiative for Asthma

^a^*N* = 4470; ^b^*N* = 4491

### Initial medications and asthma control at week 12

Overall, 90.2% of patients (4051/4491) were initially prescribed ICS/LABA, with or without other medications. Two thirds of patients (66.7%, 2997/4491) were initially prescribed ICS/LABA plus one or more additional medications, and 23.5% of patients (1054/4491) were initially prescribed ICS/LABA alone (Table 2). ICS/LABA+LTRA plus other medication(s) was the most commonly prescribed combination (40.8%, 1833/2449, Table 2).

**Table 2:** Initial medication categories

Category, n (%)	*N* = 4491
ICS/LABA	1054 (23.5)
LTRA	96 (2.1)
THO	4 (0.1)
SABA	15 (0.3)
LAMA/SAMA	4 (0.1)
ICS	8 (0.2)
Other single drug	10 (0.2)
ICS/LABA + other combined drugs^a^	2997 (66.7)
ICS/LABA + LTRA + THO + SABA + others	49 (1.1)
ICS/LABA + LTRA + THO + others	374 (8.3)
ICS/LABA + LTRA + SABA + others	200 (4.5)
ICS/LABA + LTRA + others	1833 (40.8)
ICS/LABA + THO + others	159 (3.5)
ICS/LABA + THO + SABA + others	11 (0.2)
ICS/LABA + SABA + others	100 (2.2)
ICS/LABA + others	271 (6.0)
LTRA + other combined drugs^b^	236 (5.3)
THO + other combined drugs^c^	16 (0.)
Other combined drugs	30 (0.7)

*ICS* inhaled corticosteroid, *LABA* long-acting β_2_ agonist, *LAMA* long-acting muscarinic antagonist, *LTRA* leukotriene receptor antagonist, *SABA* short-acting β_2_ agonist, *SAMA* short-acting muscarinic antagonist, *THO* theophylline

^a^Including ICS combined with LABA; ^b^Excluding ICS/LABA, ICS or LABA; ^c^Excluding ICS/LABA, ICS, LABA or LTRA

In patients prescribed ICS/LABA+LTRA versus those prescribed ICS/LABA, the proportions of patients achieving asthma control at Week 12 according to GINA 2012 criteria were 78.1% vs 80.0, 86.5% vs 85.8, 78.5% vs 71.3, and 59.6% vs 61.8% among those with intermittent, mild, moderate, and severe asthma at baseline, respectively. Across all severity levels, there were no numeric differences in the proportions of patients with any GINA 2012 control levels at Week 12 between those that had been prescribed ICS/LABA and those prescribed ICS/LABA+LTRA at baseline (Table 3).

**Table 3:** GINA 2012- and 2018-defined control level at Week 12 in patients using ICS/LABA with and without LTRA

Baseline asthma severity	Control status at Week 12	GINA 2012			GINA 2018		
		ICS/LABA + LTRA, n (%)	ICS/LABA, n (%)	Total, n (%)	ICS/LABA + LTRA, n (%)	ICS/LABA, n (%)	Total, n (%)
Intermittent	Controlled	32 (78.1)	32 (80.0)	64 (79.0)	46 (86.8)	47 (95.9)	93 (91.2)
	Partly controlled	9 (22.0)	8 (20.0)	17 (21.0)	7 (13.2)	2 (4.1)	9 (8.8)
	Uncontrolled	0 (0)	0 (0)	0 (0)	0 (0)	0 (0)	0 (0)
	Total	41	40	81	53	49	102
Mild	Controlled	122 (86.5)	121 (85.8)	243 (86.2)	167 (86.1)	178 (93.2)	345 (89.6)
	Partly controlled	18 (12.8)	19 (13.5)	37 (13.1)	24 (12.4)	11 (5.8)	35 (9.1)
	Uncontrolled	1 (0.7)	1 (0.7)	2 (0.7)	3 (1.6)	2 (1.1)	5 (1.3)
	Total	141	141	282	194	191	385
Moderate	Controlled	215 (78.5)	149 (71.3)	364 (75.4)	325 (82.1)	255 (85.3)	580 (83.5)
	Partly controlled	54 (19.7)	58 (27.8)	112 (23.2)	61 (15.4)	43 (14.4)	104 (15.0)
	Uncontrolled	5 (1.8)	2 (1.0)	7 (1.5)	10 (2.5)	1 (0.3)	11 (1.6)
	Total	274	209	483	396	299	695
Severe	Controlled	558 (59.6)	338 (61.8)	896 (60.4)	921 (71.9)	581 (77.6)	1502 (74.0)
	Partly controlled	339 (36.2)	185 (33.8)	524 (35.3)	312 (24.4)	150 (20.0)	462 (22.8)
	Uncontrolled	40 (4.3)	24 (4.4)	64 (4.3)	48 (3.8)	18 (2.4)	66 (3.3)
	Total	937	547	1484	1281	749	2030

*GINA* Global Initiative for Asthma, *ICS* inhaled corticosteroid, *LABA* long-acting β_2_ agonist, *LTRA* leukotriene receptor antagonist

Similar results were obtained when control levels were assessed using GINA 2018 criteria. In patients using ICS/LABA+LTRA compared with those using ICS/LABA, the proportions of patients achieving asthma control at Week 12 were 86.8% vs 95.9, 86.1% vs 93.2, 82.1% vs 85.3, and 71.9% vs 77.6% among those with intermittent, mild, moderate, and severe asthma at baseline, respectively. For both drug combinations, when compared with GINA 2012 criteria, GINA 2018 criteria placed a slightly greater proportion of patients from most severity categories into the controlled asthma category, with a reciprocal decrease in the proportion of patients placed in the partly controlled asthma category (Table 3).

### GINA 2012 vs GINA 2018 vs ACQ-5

**Fig. 1:**
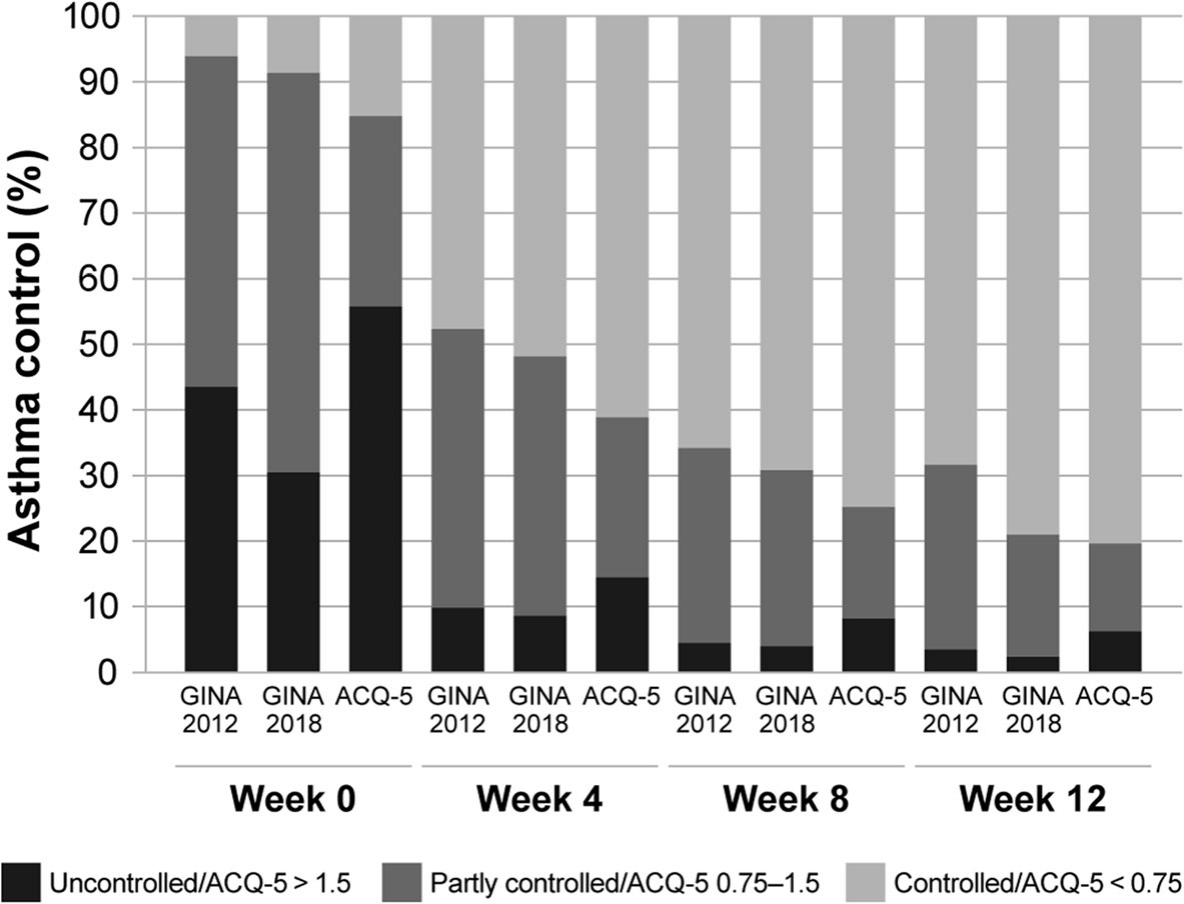
Asthma control defined by GINA 2012, GINA 2018 and ACQ-5

Across the entire population, assessment of asthma control as per GINA 2012 criteria indicated a greater proportion of patients having uncontrolled asthma and a lower proportion having controlled asthma than evaluation as per GINA 2018 criteria at every time point (Fig. 1). Assessment according to patients’ ACQ-5 scores led to a greater percentage of uncontrolled (ACQ-5 > 1.5) and controlled (ACQ-5 < 0.75) asthma than classification as per either GINA 2012 or GINA 2018 criteria at all time points (Fig. 1).

**Fig. 2:**
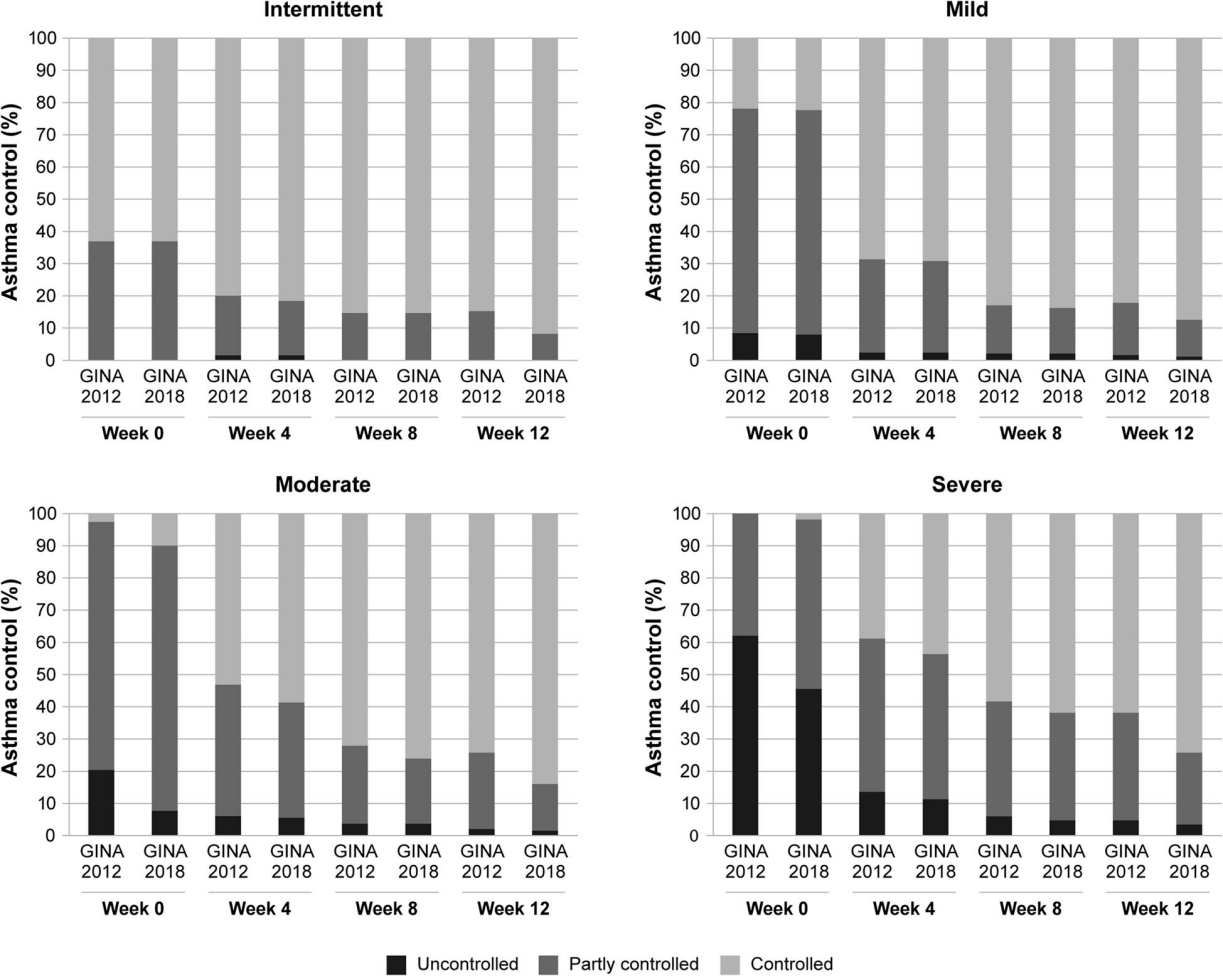
Asthma control defined by GINA 2012 and GINA 2018 according to baseline severity

When baseline severity was taken into account, asthma control rates were similar for intermittent and mild asthma patients using both classifications (Fig. 2). For patients with moderate and severe asthma, the difference between the two classification systems was more pronounced: assessment as per GINA 2012 criteria indicated a greater percentage of patients having uncontrolled asthma and a lower percentage having controlled asthma than that as per GINA 2018 criteria at every time point (Fig. 2). Irrespective of the GINA criteria used, the proportion of patients with uncontrolled and partly controlled asthma was greater in those with moderate and severe asthma than in patients with intermittent and mild persistent asthma. Furthermore, the distribution of patients between control levels was similar for both GINA 2012 and GINA 2018 criteria.

## Discussion

Both GINA 2012 criteria, in use at the time of the INITIAL study, and the GINA guidelines in place at the time of this analysis (2018) recommend low and medium−/high-dose ICS/LABA as the preferred option for patients at Step 3 and Step 4 [1, 2]. The addition of a third controller (LTRA or theophylline) is an option for patients who are not adequately controlled with a medium-dose ICS/LABA after a trial of 2–3 months for patients at Step 4 [1, 2]. Among ICS/LABA medications, budesonide/formoterol is recommended by GINA 2019 as the preferred formulation of ICS-formoterol at step 1 and 2 based on the evidence of efficacy, and it was also the predominant form used in our population at baseline (88.9%, 3602/4051) [13], probably because of their availability in one inhaler (e.g. Symbicort) and easy inhaler techniques. Despite these recommendations, ICS/LABA+LTRA (plus others) was the most commonly prescribed initial therapy in 40.8% (1833/4491) of patients, and only 23.5% (1054/4491) of patients were prescribed ICS/LABA only at baseline. Furthermore, there was no numeric difference in the number of patients achieving asthma control at Week 12, irrespective of whether they had been prescribed ICS/LABA and ICS/LABA+LTRA at baseline and regardless of their initial severity level. This suggests that LTRA may have been prescribed unnecessarily in some patients in the INITIAL study. The number of medications prescribed and the cost and complexity of a treatment regimen may also have a negative impact on adherence [2, 17]. As medication costs form the largest component of direct medical expenditures in asthma [18], additional medications place further economic burden on patients and healthcare systems. Studies have shown that patients who adhere to their treatment regimen have better control of their asthma [19].

Despite the lack of clinical benefits with LTRA observed among asthmatic patients in general, it may be a preferred choice for the subset of asthma patients with concomitant allergic rhinitis. Leukotriene plays a critical role in nasal vascular permeability, mucus production, and hypersecretion upon allergen provocation in allergic rhinitis [20, 21]. By inhibiting the pathogenic process, LTRA is a promising class of medication that can relieve symptoms of rhinitis. Improvement of rhinitis symptoms by LTRA has been demonstrated by a multicentre, placebo-controlled study in patients with symptomatic allergic rhinitis and active asthma (difference between montelukast and placebo in mean change in Daily Rhinitis Symptoms score from baseline, − 0.12 [95% CI, − 0.18 to − 0.06; *p* < or = 0.001]) [10]. The efficacy and benefits of LTRA in allergic rhinitis was also supported by results of a meta-analysis study, in which LTRA reduced mean daily rhinitis symptom scores (by 5% [95% CI, 3–7%]) and improved rhinoconjunctivitis quality of life (by 0.3 [95% CI, 0.24–0.36]) [22]. LTRA (HDM SLIT in particular) has been recommended by GINA 2019 as an add-on medication for patients with allergic rhinitis and FEV_1_ > 70% at step 3 and 4 [23]. However, in the INITIAL study, patients with an allergic history only comprised 22.1% of the patient population [13]. Although these patients might derive benefits from LTRA, the majority of asthmatic patients without allergic rhinitis (77.9%) still required more effective medications, such as ICS plus LABA, for symptom control and exacerbation prevention [2].

Collection of lung function data was at the discretion of the investigator because lung function testing is not a mandatory requirement of the Chinese Thoracic Society Guidelines 2008 [14]. Therefore, lung function data were not collected for 2942, 2947 and 964 patients at Weeks 4, 8 and 12, respectively, and so GINA 2012 control level could not be determined in these patients. As a result, patient numbers for the comparison of ICS/LABA+LTRA versus ICS/LABA at Week 12 were low, representing only one third (1484) of the FAS. In 2014, GINA criteria underwent a major revision that changed the determination of symptom control by removing lung function testing from the assessment criteria [3]; these criteria have remained unchanged in subsequent reports including the 2018 version [2]. Lung function is now considered, along with exacerbations, as a risk factor for poor asthma outcome [3].

Juniper et al. originally developed the ACQ as a seven-item measure that included forced expiratory volume in 1 s (FEV_1_) [9]. In their 2006 paper, they suggest that while the cut-off point between ‘well-controlled’ and ‘not well-controlled’ asthma is close to 1.00, to be confident that patients are categorised correctly in clinical practice, the optimal cut-offs for well-controlled asthma and inadequately controlled asthma should be 0.75 and 1.50, respectively [24]. GINA 2006-defined asthma control (that includes lung function) has been shown to have reasonable agreement with the ACQ-5 (that does not include lung function) cut-off points [25]. GINA 2006 controlled, partly controlled and uncontrolled patients had mean ACQ-5 scores of 0.43, 0.75 and 1.62, respectively [25]. Another study of the ACQ by Sastre et al. suggested that the cut-off differs depending on whether lung function is included (ACQ without lung function, equivalent to ACQ-5: 0.83) and depending on which test is used (ACQ with FEV_1_, equivalent to ACQ-7: 1.14; ACQ with peak expiratory flow [PEF]: 1.28) [26]. However, a more recent study suggested that the cut-off for uncontrolled asthma was 1.00, despite suggesting a similar cut-off for controlled asthma of 0.50 for both the ACQ-5 (that does not include lung function) and the ACQ-7 (that includes lung function) [27]. In keeping with these findings, the results of the present post hoc analysis suggest that, in this data set, the removal of lung function test results from the GINA 2014 and later criteria reduces the number of patients considered to have uncontrolled asthma and increases the number considered to have controlled asthma. When baseline severity was taken into account, this difference appeared to be driven by patients with moderate and severe asthma. However, control levels did not differ a great deal between the two classifications, supporting the view that lung function does not correlate strongly with asthma symptoms [28, 29].

## Conclusions

Over 40% of patients in the INITIAL study were prescribed ICS/LABA+LTRA; however, in the patients available for analysis at Week 12, this did not alter the levels of GINA 2012- or 2018-defined asthma control compared with ICS/LABA combination alone. While stepping up treatment to include additional controllers is a valid approach after a preliminary trial with a new regimen, the addition of LTRA to ICS/LABA as the initial treatment may have been unnecessary in some of these newly diagnosed patients. Physicians should follow GINA and other guidelines by initiating treatment for patients at Step 3 and Step 4 with ICS/LABA for maintenance and relief and then wait 2–3 months before considering stepping up treatment and/or adding additional controllers.

## Data Availability

The datasets used and/or analysed during the current study are available from the corresponding author on reasonable request.

## Abbreviations

ACQ-5: Asthma Control Questionnaire (5-item version)
FAS: Full analysis set
FEV_1_: Forced expiratory volume in 1 s
GINA: Global Initiative for Asthma
ICS: Inhaled corticosteroids
ICS/LABA: Inhaled corticosteroid/long-acting β_2_ agonist
LTRA: Leukotriene receptor antagonist
PEF: Peak expiratory flow
